# Step-by-Step Description of Standardized Technique for Robotic Pancreatoduodenectomy

**DOI:** 10.3390/curroncol32060302

**Published:** 2025-05-24

**Authors:** Antonella Delvecchio, Silvio Caringi, Cataldo De Palma, Gaetano Brischetto, Rosalinda Filippo, Annachiara Casella, Valentina Ferraro, Matteo Stasi, Riccardo Memeo, Michele Tedeschi

**Affiliations:** 1 Unit of Hepato-Biliary and Pancreatic Surgery, F. Miulli General Hospital, Acquaviva delle Fonti, 70021 Bari, Italy; silviocaringi@hotmail.it (S.C.); cataldo.depalma@st.hunimed.eu (C.D.P.); gaetano.brischetto@studenti.univr.it (G.B.); r.filippo@miulli.it (R.F.); a.casella@miulli.it (A.C.); v.ferraro@miulli.it (V.F.); matteo.stasi@miulli.it (M.S.); r.memeo@miulli.it (R.M.); m.tedeschi@miulli.it (M.T.); 2 Department of Surgery, Università Degli Studi Roma Tor Vergata, 00133 Rome, Italy; 3 Pancreatic Surgery Unit, IRCCS Humanitas Research Hospital, Rozzano, 20089 Milan, Italy; 4 Pancreatic Surgery Unit, University of Verona, 37134 Verona, Italy; 5 Department of Medicine and Surgery, LUM University, Casamassima, 70010 Bari, Italy

**Keywords:** Whipple procedure, minimally invasive surgery, robotic pancreaticoduodenectomy, standardization, surgical technique, reproducibility

## Abstract

Robotic pancreaticoduodenectomy (RPD) has emerged as a viable alternative to open and laparoscopic approaches, offering potential advantages in precision and dexterity. However, its complexity and lack of standardization remain as barriers to widespread adoption. We present a step-by-step surgical approach to RPD, emphasizing key technical strategies to enhance safety, efficiency, and reproducibility. Our technique is structured into defined surgical steps, facilitating learning curve optimization and intraoperative consistency. Key refinements include an optimized trocar placement, the strategic suspension of vascular structures, and specific reconstructive techniques to reduce the operative time and improve surgical ergonomics. These improvements may contribute to a reduction in perioperative morbidity and procedural standardization. Standardizing RPD through defined surgical steps and structured learning pathways may improve its feasibility, safety, and broader adoption. Further studies are needed to validate these strategies in high-volume centers.

## 1. Introduction

Pancreaticoduodenectomy (Whipple procedure) is a highly complex surgical intervention performed for malignant and benign diseases of the pancreas, duodenum, and bile ducts. It involves a dissection phase, requiring a meticulous dissection near critical vascular structures, followed by a reconstructive phase consisting of three anastomoses. Due to its complexity, achieving proficiency in this procedure requires extensive surgical experience and technical expertise. Minimally invasive approaches, including laparoscopic and robotic techniques, have been developed to reduce surgical trauma, minimize blood loss, and enhance the postoperative recovery [1,2,3,4]. The laparoscopic pancreaticoduodenectomy (LPD), first reported by Gagner and Pomp in 1994 [5], has not gained widespread adoption due to technical complexity, prolonged operative times, two-dimensional visualization, restricted instrument maneuverability, and a steep learning curve [6,7,8]. In contrast, robotic pancreaticoduodenectomy (RPD), introduced by Giulianotti in 2001 [9], has emerged as a promising alternative, offering key advantages such as three-dimensional visualization, wristed instruments, and a greater precision in dissection and reconstruction [10,11,12]. Emerging evidence suggests that RPD may improve lymph node yield, margin-negative resection rates (particularly in pancreatic adenocarcinoma), and reduce intraoperative blood loss compared to open and laparoscopic approaches [13,14,15]. However, despite these benefits, RPD remains a technically demanding procedure requiring specialized training, high surgical expertise, and standardized techniques to ensure safety and reproducibility. Standardization remains elusive, with significant variability in anastomotic methods, trocar placement, and patient selection criteria. This heterogeneity underscores the need for systematic protocols to ensure reproducibility and safety, particularly during the learning curve. This article provides a comprehensive, step-by-step approach to RPD, outlining key technical aspects, procedural refinements, and practical “tips and tricks” aimed at enhancing surgical safety, improving learning curve efficiency, and promoting standardization in robotic pancreatic surgery.

## 2. Methods

**Figure 1:**
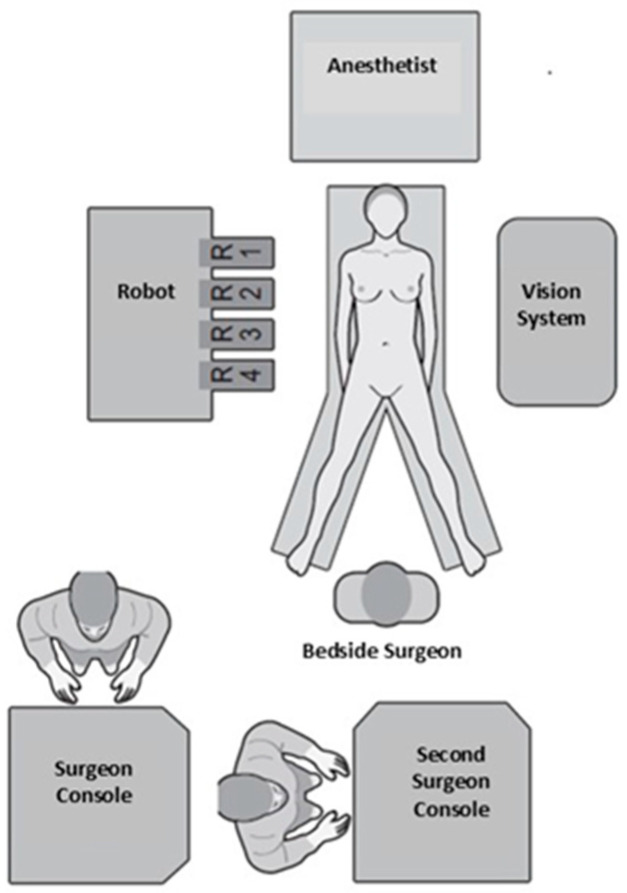
Operating room layout.

We present our standardized step-by-step surgical technique for RPD. The operative steps are outlined in Table 1, with the dissection phase consisting of 10 steps and the reconstructive phase of 5 steps. Additionally, standardizing surgical instruments (Table 2) and the operating room layout (Figure 1) is essential for both the surgeon and the nursing staff. This organization helps minimize the setup time, reduce downtime in the operating room, and eliminate stress related to missing instruments, particularly during the critical phases of the procedure.

**Table 1:** Operative steps of RPD.

Demolitive Phase
Exposure Taking down hepatic flexure Extensive Kocher maneuver First jejunal loop transection Gastrocolic ligament division Hepatic hilum dissection Cholecystectomy and bile duct transection Stomach transection Pancreas transection Specimen mobilization
**Reconstructive phase**
11. Pancreaticojejunostomy 12. Hepaticojejunostomy 13. Gastrojejunostomy 14. Drain placement 15. Specimen removal

**Table 2:** List of instruments and role.

Robotic Xi Platform	
Maryland Bipolar Forceps	Precise dissection and coagulation
Fenestrated Bipolar Forceps	Grasping and coagulation
Tip-up Fenestrated Grasper	Gentle tissue grasping
Monopolar Curved Scissors	Cutting and dissecting tissue
Needle Driver	Suturing during anastomoses
Vessel Sealer	Sealing and dividing blood vessels
Laparoscopic and Robotic Clip Applier	Applying clips to blood vessels or ducts (e.g., Hem-o-lok clips)
Stapling Devices	Linear staplers for transecting the stomach, jejunum, or other structures
Laparoscopic Graspers	Retracting, manipulating, and holding tissues
Laparoscopic Suction–Irrigation Device	Clearing the surgical field of blood and debris
Specimen Retrieval Bag	Removing the resected specimen from the abdominal cavity
Drain	

### 2.1. Operating Room Layout

Under general anesthesia, the patient is placed in a supine position with the legs spread (French position), while the bedside surgeon stands between the patient’s legs. The robotic console is positioned to the patient’s right and the vision system to the left. The dual console is placed at the back of the room, beyond the patient’s legs, facilitating seamless communication between the two console surgeons and the bedside surgeon (Figure 1).

### 2.2. Trocar Placement and Patient Positioning

**Figure 2:**
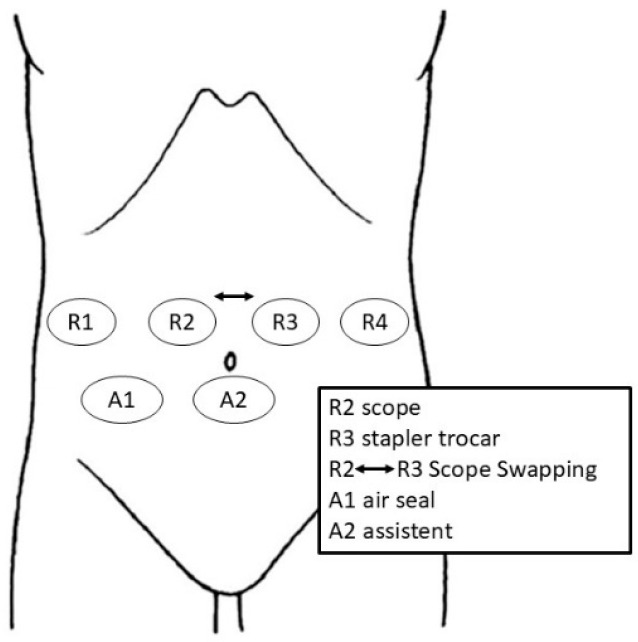
Trocar placement.

Trocar ports are strategically positioned to optimize access for the robotic arms and assistant instruments. Typically, six ports are used: four for the robotic system and two for laparoscopic assistance (Figure 2). After creating pneumoperitoneum (12 mmHg) using the open technique in the R3 position, a 12.8 mm reducer trocar is placed to the left and above the umbilicus for the stapler and/or camera. A diagnostic laparoscopy is then performed to exclude liver metastases or carcinomatosis.

The remaining ports are inserted under direct vision for safety, maintaining an 8–10 mm distance between them. An 8 mm trocar (R2) is placed on the right side above the umbilicus. The R1 port is positioned along the right anterior axillary line, while R4 is placed symmetrically on the left anterior axillary line. A laparoscopic trocar (A1) is placed above the umbilicus for the AirSeal system, and an additional assistant port (A2) is positioned in the right iliac fossa.

**Figure 3:**
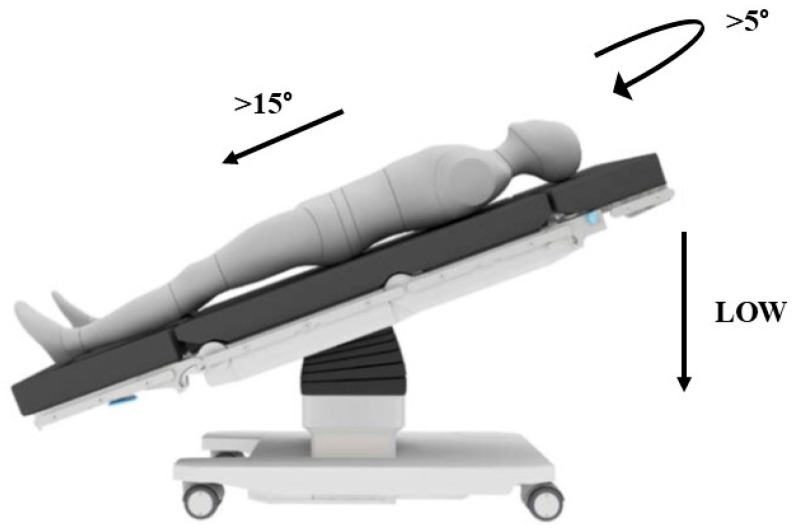
Patient positioning.

After port placement, the operating table is adjusted to a reverse Trendelenburg position (>15°) with a slight left tilt (>5°) and positioned as low as possible (Figure 3).

The robotic cart is docked head-on. Intraoperatively, the camera position is interchanged between R2 and R3 to optimize visualization. For instance, the camera is positioned in R2 during the hilum lymphadenectomy, Kocher maneuver, and hepaticojejunostomy, while it is placed in R3 during the pancreas dissection and transection. 

## 3. Operative Steps

### 3.1. Dissection Phase

#### 3.1.1. Step 1: Exposure

**Figure 4:**
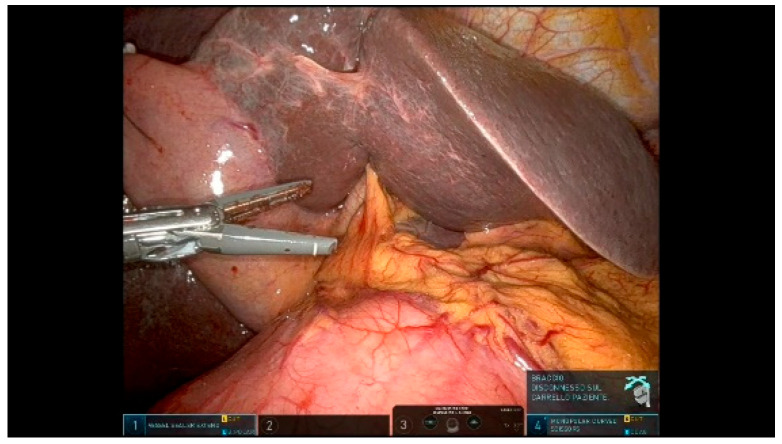
Exposure.

In this phase, the camera is positioned in R2. The gallbladder fundus and round ligament are anchored to the parietal peritoneum, allowing for the liver retraction and optimal exposure of the surgical field. This technique frees the fourth robotic arm for additional tasks, enhancing surgical efficiency (Figure 4).

#### 3.1.2. Step 2: Taking Down Hepatic Flexure

The right colonic flexure is taken down along the plane between Toldt’s and Gerota’s fascia to fully expose the descending segment of the duodenum. Mobilization is carried out up to the origin of the transverse mesocolon. The Monopolar Curved Scissors in R1 and the Tip-up Fenestrated Grasper in R2 are used for dissection, while R4 is utilized for the exposure of anatomical structures.

#### 3.1.3. Step 3: Extensive Kocher Maneuver

Extensive Kocherization is performed to fully expose the inferior vena cava, left renal vein, and aorta. The Treitz ligament is divided, and the complete detachment of the pancreatic head from the retroperitoneal space is achieved, allowing for a safer and more efficient uncinate process dissection. The maneuver also facilitates an accurate assessment of the tumor resectability. Para-aortic lymph node (LN 16) sampling is performed for a frozen analysis, and the procedure continues only if no neoplastic infiltration is detected. Specimens for the frozen section is retrieved using a sterile glove finger introduced through the assistant trocar. During this phase, the Vessel Sealer in R4 is used to retract the duodenum to the left, fully mobilizing the descending portion of the duodenum and the dorsal part of the pancreatic head. The Fenestrated Bipolar Forceps are placed in R1, while the Monopolar Curved Scissors are positioned in R3.

#### 3.1.4. Step 4: First Jejunal Loop Transection

**Figure 5:**
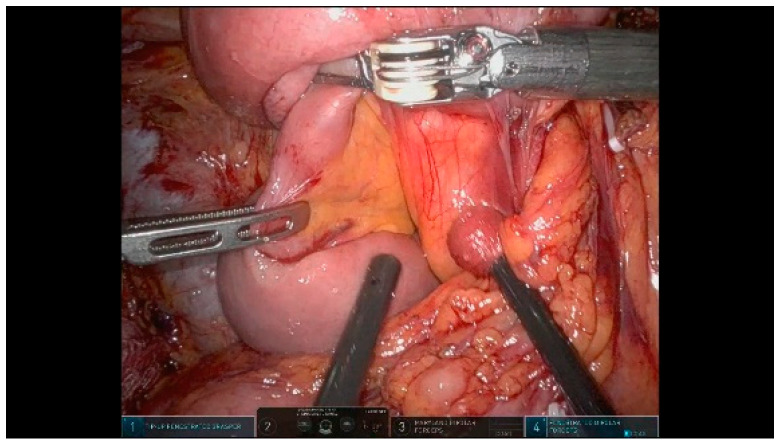
First jejunal loop transection.

The first jejunal loop is retracted to the right of the aortomesenteric axis using a Tip-up Fenestrated Grasper in R1 and divided with a robotic stapler via the posterior part of the superior mesenteric vein (SMV), (Figure 5). A complete derotation of the duodenojejunal flexure and the detachment of the uncinate process is achieved. The jejunal mesentery is dissected close to the jejunal side using a Vessel Sealer in R3 to minimize the risk of bleeding from mesenteric vessels.

#### 3.1.5. Step 5: Gastrocolic Ligament Division

Using the R4 robotic arm, the stomach is lifted upward with a Tip-up Fenestrated Grasper to apply appropriate tension on the gastrocolic ligament. The gastrocolic ligament is divided using the Vessel Sealer in R3, allowing entry into the lesser sac. This provides access to the anterior surface of the pancreas and the posterior gastric wall. The right gastroepiploic vessels are carefully dissected, ligated, and divided.

#### 3.1.6. Step 6: Hepatic Hilum Dissection

The dissection of the hepatic hilum is performed to expose the common hepatic artery (CHA), proper hepatic artery (PHA), portal vein (PV), and common bile duct (CBD). A thorough vascular assessment is performed to identify any anatomical variants. The dissection begins from the CHA, proceeding from left to right. Not all lymph nodes can be harvested en bloc with the specimen. To facilitate exposure and enhance the lymphadenectomy, vessels are suspended using a vessel loop with the R4 arm.

**Figure 6:**
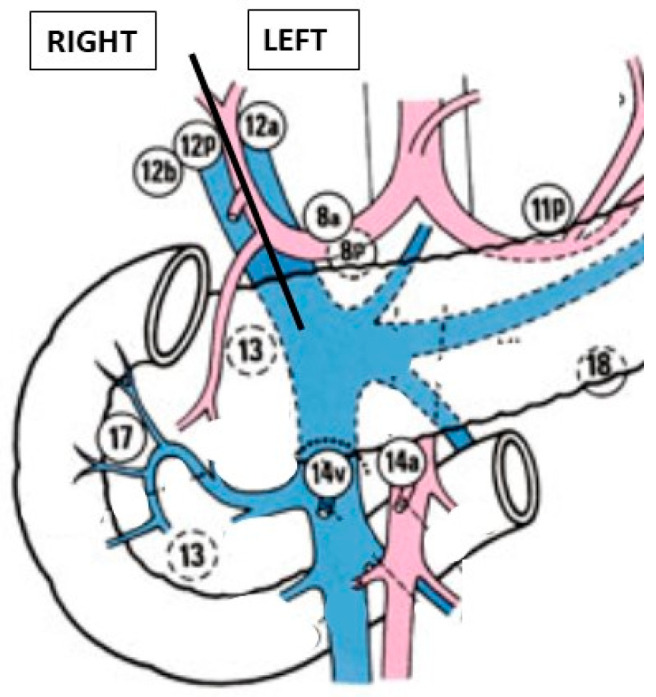
Lymph nodes.

The supra-pancreatic triangle (bounded by the gastroduodenal artery [GDA], CHA, and superior pancreatic margin) is dissected to expose the anterior wall of the PV. The right gastric artery is identified, ligated, and divided. Lymph nodes within the hepatoduodenal ligament are separated into left and right groups along the plane defined by the PHA and PV (Figure 6):

Right side: 12b and 12p
Left side: 12a, 8a, and 8p

The left-sided lymph nodes are dissected and retrieved laparoscopically using a glove finger for extraction. The right-sided lymph nodes are dissected and removed en bloc with the specimen. All soft tissues surrounding the hepatoduodenal ligament are thoroughly dissected and the structures are skeletonized to ensure an optimal oncological clearance.

**Figure 7:**
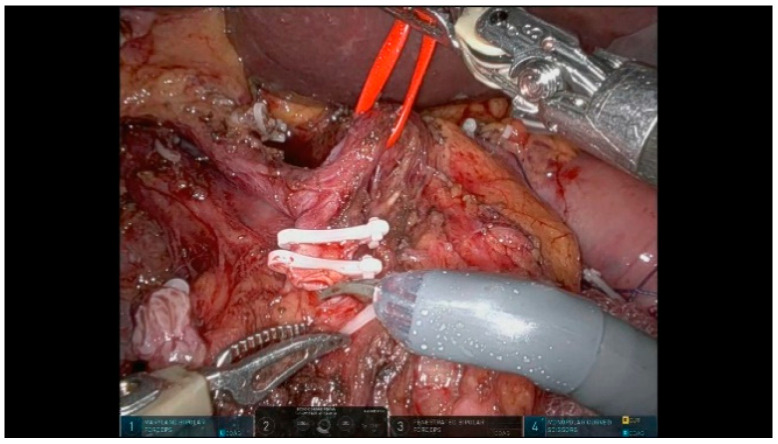
GDA division.

The GDA is divided between Hem-o-lok clips after confirming a preserved hepatic arterial inflow (Figure 7).

#### 3.1.7. Step 7: Cholecystectomy and Bile Duct Transection

The gallbladder artery and cystic duct are carefully dissected, ligated, and divided. However, the gallbladder itself is not removed at this stage to maintain adequate exposure.

The CBD is divided above the cystic duct insertion using cold scissors, and the distal duct is clamped with a Hem-o-lok clip. In cases of distal cholangiocarcinoma, the histological biliary margin is sent for an intraoperative frozen section analysis to assess the tumor involvement. The specimen for the frozen section is retrieved using a sterile glove finger introduced through the assistant trocar.

#### 3.1.8. Step 8: Stomach Transection

The vascular transection of the right gastroepiploic and right gastric arteries has already been performed. The stomach is suspended using a resorbable suture at the level of the transection to expose the posterior wall and ensure the proper alignment of the robotic stapler. The distal stomach is transected approximately 3 cm proximal to the pylorus using a robotic stapler. Before firing, the stapler is kept closed for a sufficient period to allow an adequate compression of the gastric wall, minimizing the risk of bleeding from the gastric stump.

#### 3.1.9. Step 9: Pancreas Transection

**Figure 8:**
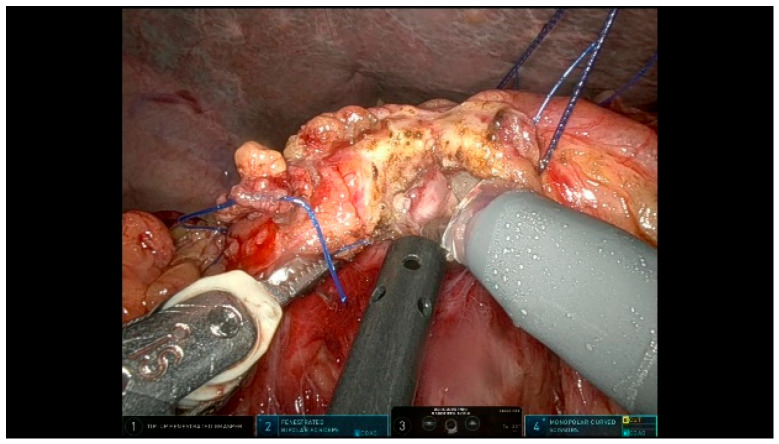
Pancreas suspension.

**Figure 9:**
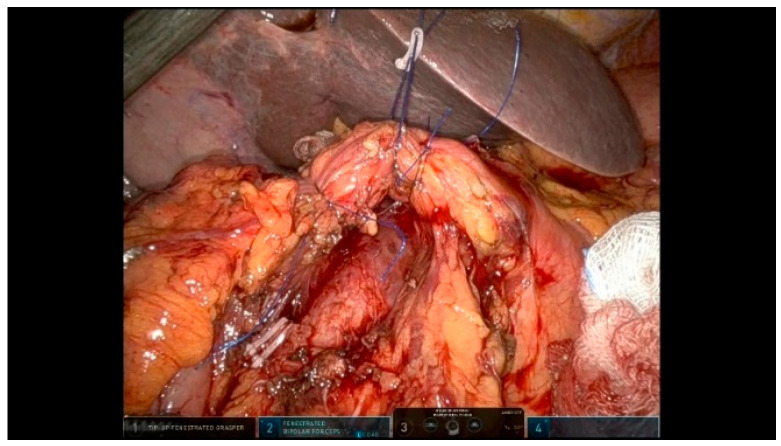
Pancreas transection.

At this stage, the camera is shifted to R3 for optimal visualization. A precise exposure of both the inferior and superior margins of the pancreatic neck is essential. Before transecting the pancreatic head, a tunnel is created anterior to the PV. The pancreas is suspended with the R4 arm using a resorbable suture, to facilitate a safe dissection. To prevent bleeding, 3-0 polypropylene sutures are placed on the upper and lower pancreatic vascular arches before the transection (Figure 8). The pancreatic neck is transected using Monopolar Curved Scissors in R3, following the anterior plane of the PV. During the parenchymal transection, the main pancreatic duct (MPD) must be carefully identified and cut with cold scissors (Figure 9). A section of the pancreatic transection margin is sent for an intraoperative frozen histological analysis. The specimen for the frozen section is retrieved using a sterile glove finger introduced through the assistant trocar. To facilitate the identification in subsequent steps, the MPD is temporarily cannulated with a silicone tube.

#### 3.1.10. Step 10: Specimen Mobilization

In this phase, the camera is shifted to R2. This is the most challenging step, involving the mobilization of the specimen by dissecting along the superior mesenteric artery (SMA), SMV, and PV.

**Figure 10:**
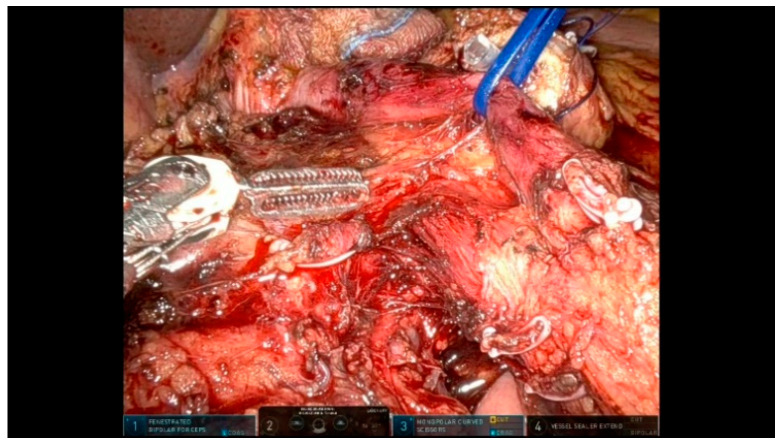
Specimen mobilization.

We typically perform a “hanging maneuver” by placing a vessel loop around the SMV. The R4 arm gently retracts the SMV laterally to the left, optimizing the exposure and facilitating the dissection of the attachments to the mesenteric vessels (Figure 10). The dissection of the SMA and SMV/PV from the uncinate process is carried out in a caudal-to-cranial direction. The inferior and superior pancreaticoduodenal vessels are dissected and divided between Hem-o-lok clips to achieve the optimal exposure of the right wall of the SMA and SMV/PV. Smaller caliber branches arising from the SMA and SMV are similarly clamped and divided using Hem-o-lok clips or the Vessel Sealer. After completing the dissection, the specimen is placed into an Endobag but remains in the abdominal cavity at this stage.

### 3.2. Reconstructive Phase

The jejunal loop is transposed through the “Treitz Window,” where the fourth portion of the duodenum normally crosses the mesocolic root (retromesenteric route) into the supramesocolic compartment to facilitate digestive reconstruction.

#### 3.2.1. Step 11: Pancreaticojejunostomy

**Figure 11:**
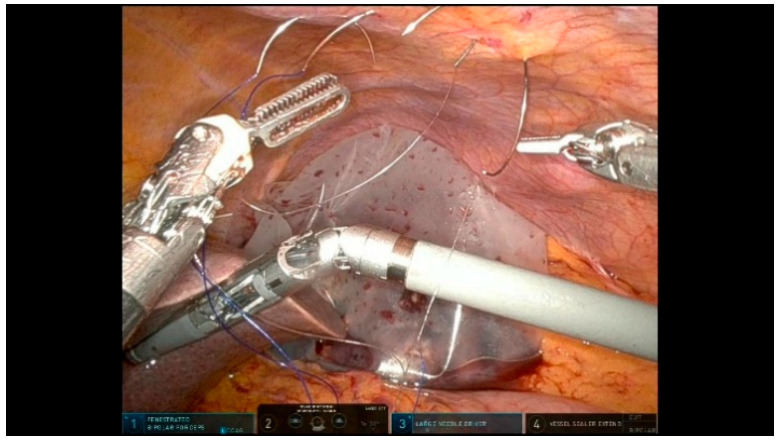
Pancreaticojejunostomy sutures.

**Figure 12:**
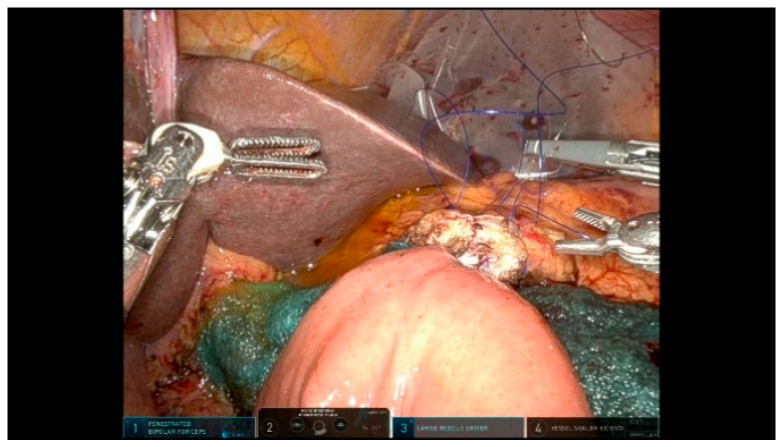
Pancreaticojejunostomy.

The Blumgart-style pancreaticojejunostomy (PJ) technique utilizes trans-pancreatic sutures to invaginate the small bowel around the pancreatic parenchyma, creating an end-to-side duct-to-mucosa anastomosis on the anti-mesenteric border. Three trans-pancreatic, interrupted, double-needle sutures using 3/0 polypropylene are placed through the full thickness of the pancreas to reduce the tension on the duct-to-mucosa anastomosis. These sutures are anchored to the parietal peritoneum, using two blue and one transparent suture alternately to prevent snagging (Figure 11). A temporary silicone tube is inserted into the MPD while placing the trans-pancreatic sutures to prevent duct injury. A small opening is created in the jejunal mucosa, and the interrupted 6/0 PDS sutures for the duct-to-mucosa anastomosis are placed in a clock-like orientation, with up to eight sutures depending on the size of the Wirsung duct. After securing the posterior wall, a definitive resorbable tube is positioned to protect the duct-to-mucosa anastomosis (Figure 12). Finally, the trans-pancreatic 3/0 polypropylene sutures are repositioned on the intestinal loop to invaginate the pancreatic parenchyma into the jejunum.

#### 3.2.2. Step 12: Hepaticojejunostomy

**Figure 13:**
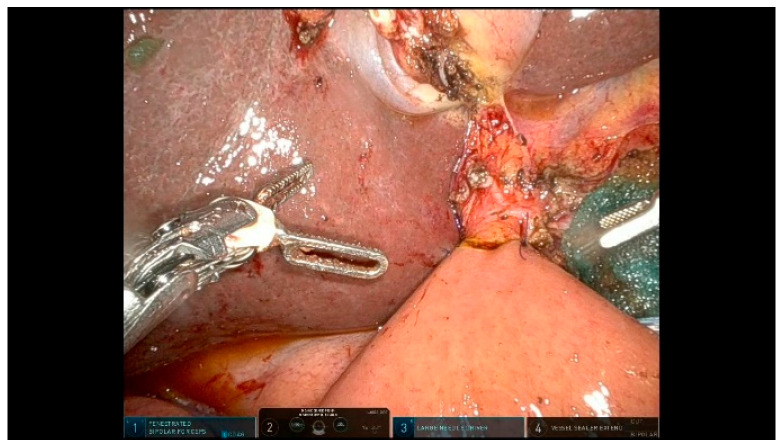
Hepaticojejunostomy.

The second anastomosis is performed 10 cm distal to the PJ (Figure 13). Generally, hepaticojejunostomy (HJ) is constructed using a single posterior and anterior layer. For bile ducts less than 10 mm in diameter, both the posterior and anterior layers are sutured with interrupted 5/0 PDS stitches. For bile ducts larger than 10 mm, the posterior layer is performed with a running 5/0 PDS suture, while the anterior layer is sutured with interrupted stitches.

#### 3.2.3. Step 13: Gastrojejunostomy

At 60 cm distal to the HJ anastomosis, an antecolic, side-to-side gastrojejunostomy (GJ) is created on the posterior wall of the stomach. This anastomosis is performed using a robotic stapler in an iso-peristaltic fashion. The stapler entry sites are closed with barbed sutures.

#### 3.2.4. Step 14: Drain Placement

Two 19 Fr silicone drains are placed:

The first drain is positioned posteriorly to the P-J and H-J and is externalized through the R1 position.

The second drain is placed anteriorly to the P-J and posteriorly to the G-J, externalized through the R4 position.

#### 3.2.5. Step 15: Specimen Removal

The specimen is extracted through a small suprapubic Pfannenstiel incision.

After removal, the pneumoperitoneum is re-established to verify the correct positioning of the drains.

Finally, the robotic system is undocked, and the trocar sites are closed.

## 4. Discussion

Pancreaticoduodenectomy, also known as the Whipple procedure, is a complex surgical intervention used to treat diseases affecting the pancreas, duodenum, and distal bile duct. The procedure involves both a dissection phase and a reconstructive phase and is associated with a high rate of postoperative morbidity.

The demanding dissection phase involves dissection in close proximity to vital vascular structures. The reconstructive phase entails the creation of three anastomoses to restore digestive function: pancreaticojejunostomy or pancreatico-gastrostomy, hepaticojejunostomy, and gastrojejunostomy.

Minimally invasive approaches, including both laparoscopic and robotic techniques, have emerged as an alternative to open surgery, offering potential benefits such as reduced blood loss, shorter hospital stays, and faster recovery [1].

A laparoscopic pancreaticoduodenectomy was first performed by Gagner and Pomp in 1994 [5].

The most recent systematic review [16], which includes four randomized controlled trials comparing an open and laparoscopic pancreaticoduodenectomy, concluded that in experienced hands and in high-volume centers, the laparoscopic approach is as feasible and safe as the open approach. However, the LEOPARD-2 trial [3] was prematurely terminated due to safety concerns regarding LPD.

Despite the potential benefits, LPD has not achieved widespread adoption, primarily due to its technical complexity, particularly during the reconstructive phase. Additionally, the steep and prolonged learning curve has discouraged many surgeons from implementing this technique [6,7].

The robotic approach has gained popularity over the past decade, benefiting from technological advancements such as three-dimensional visualization, wristed instruments, tremor filtration, enhanced precision, and increased dexterity. These advantages help overcome the limitations of the laparoscopic approach, particularly in pancreatic surgery [10,12].

The robotic pancreatoduodenectomy is a complex procedure that should be performed by highly trained surgeons in specialized centers with expertise in both pancreatic surgery and robotic techniques [10,17].

The first RPD was performed by Giulianotti in 2001 [5], but its complexity and lack of standardization have hindered its widespread adoption.

Retrospective studies suggest that RPD is safe, feasible, and a valid alternative to the open approach [1,10,17].

The EUROPA trial [18] represents a significant effort to compare RPD and open pancreaticoduodenectomy (OPD). The study concluded that, in high-volume centers with trained surgeons, both RPD and OPD are safe procedures, without significant differences in intraoperative blood loss, wound complications, or hospital stay.

After initial experiences, RPD has gained broader acceptance. However, major obstacles to its wider adoption include the limited availability of robotic platforms, the lack of surgical standardization, high costs, and the challenges of technique acquisition.

The learning curve in minimally invasive surgery refers to the gradual improvement in surgical proficiency, efficiency, and patient outcomes as a surgeon gains experience with the procedure. Studies suggest that achieving proficiency in RPD requires between 33 and 80 cases, depending on the surgeon’s prior experience, training environment, and institutional support. In contrast, LPD has a steeper learning curve, with a recommended minimum of 50 cases to achieve surgical competence. The broader range for RPD is attributed to variations in prior laparoscopic experience and robotic skill acquisition [19,20].

Here, we present our step-by-step surgical experience with RPD, emphasizing key practical “tips and tricks” that may facilitate safe adoption and technique standardization.

Several centers have proposed stepwise approaches to RPD in an effort to standardize this complex procedure. Giulianotti et al. [8] described a 17-step technique focusing on anatomical segmentation and progressive skill acquisition, emphasizing the importance of a structured learning path for robotic training. Giulianotti also highlighted key technical challenges, such as the extended Kocher maneuver, uncinate process dissection, and anastomotic complexity, as specific steps requiring greater attention within a modular framework. Zhao et al. [21] expanded the concept by defining an 18-step protocol, underlining the value of technical simplification to enhance reproducibility.

Our technique aligns with these methodologies in aiming to facilitate reproducibility, reduce variability, and improve educational value through standardization.

However, our method introduces several distinctive features that differentiate it from existing protocols.

We have structured our technique into surgical steps categorized by different levels of difficulty. This approach can be beneficial during the learning curve, allowing each step to be performed by surgeons with varying degrees of expertise. Standardizing the surgical steps ensures a uniform execution by different surgeons and enhances the teaching process by focusing on specific anatomical landmarks. In this context, the implementation of a standardized, step-by-step surgical protocol plays a crucial role in reducing variability and facilitating progressive skill acquisition. Our 15-step technique was specifically designed not only to enhance intraoperative reproducibility, but also to serve as a structured framework for phased training strategies, including modular teaching, simulation-based learning, and dual-console mentoring.

Each surgical step corresponds to a distinct anatomical or technical goal, which allows for the targeted training of junior surgeons and supports a gradual team integration. This structured approach has been shown to improve operative performance and may significantly shorten the learning curve, both for individual surgeons and for multidisciplinary robotic teams establishing a new RPD program.

The following tips and tricks are designed to enhance efficiency, improve visualization, and ensure safety throughout the procedure.

Tips and Tricks

- Trocar Placement: ensure optimal trocar positioning based on the patient size and anatomy to facilitate smooth instrument movement and minimize external collisions between robotic arms.
- Pneumoperitoneum Management: use an AirSeal system to maintain a stable pneumoperitoneum and improve visualization, especially during prolonged dissection phases.
- Double Assistant Trocar: Utilizing two assistant trocars improves the workflow efficiency. One dedicated to suction and irrigation, the other for assisting with exposure, introducing gauze, or passing needles during reconstruction. This setup helps reduce the operative time and enhances surgical precision.
- Liver Retraction: anchor the gallbladder and round ligament to the abdominal wall to free the fourth robotic arm for other tasks.
- Jejunal Resection: perform the jejunal division at the end of an extended Kocher maneuver to facilitate orientation.
- Suspension Techniques: Use resorbable loops or vessel loops to suspend organs and vascular structures, optimizing exposure and safety. These loops help retract and define key landmarks, such as the SMV, without excessive manipulation.
- Pancreatic Neck Transection: ensure the perfect exposure of the inferior and superior pancreatic margins before transection; use vessel loops for better control.
- “Hanging Maneuver”: place a vessel loop around the SMV to facilitate a safe uncinate dissection process.
- Duct-to-Mucosa Anastomosis: use a temporary silicone stent to protect the pancreatic duct and facilitate precise suturing.
- Suture Organization: use different colored sutures to prevent tangling and enhance efficiency during anastomoses.
- Drain Placement: position drains strategically to prevent fluid accumulation and improve postoperative outcomes, particularly near the pancreatojejunostomy and hepaticojejunostomy sites, which can assist in the early identification of potential leaks.

Although the proposed 15-step standardized technique provides a reproducible and structured approach to RPD, we acknowledge that it may not be universally applicable in an identical fashion to all patients. Clinical and anatomical variability, such as the degree of obesity, local inflammation, preoperative chemotherapy, or the vascular involvement of the SMV, PV, or SMA, may require tailored intraoperative strategies. In our experience, the standardized technique serves as a solid reference that can be adapted intraoperatively based on patient-specific conditions. For example, in cases with extensive visceral fat or a narrow working space, port positioning and retraction strategies are adjusted accordingly. When major vessel involvement is suspected, an artery-first approach is preferred to ensure the clear identification and control of the vascular structures. Additionally, when a vascular resection is necessary, we sometimes prefer to adopt an open approach, as it allows for more precise control and a better view of the involved vessels, ensuring safety during complex dissections. Furthermore, all cases are planned preoperatively to anticipate anatomical variations and potential technical challenges.

While our current work focuses on describing the surgical technique, future research will be directed towards evaluating clinical outcomes, perioperative results, and the impact of stepwise standardization on the learning curve. Moreover, further studies will be needed to explore the oncological outcomes and long-term survival rates associated with our standardized RPD approach.

We believe that sharing detailed technical descriptions is essential to facilitating the broader adoption of RPD and to inspiring multicentric collaborations aimed at validating and refining standardized protocols.

## 5. Conclusions

RPD remains a challenging procedure with prolonged operative times, high technical demands, a steep learning curve, and significant postoperative morbidity. Standardizing the technique can optimize procedural efficiency, shorten the learning curve, reduce operative times, and consequently lower postoperative morbidity.

Randomized trials are still needed to confirm whether robotic surgery offers significant advantages over the open approach.

## Data Availability

No data were generated or analyzed in this study.

## Abbreviations

RPD	Robotic pancreaticoduodenectomy
LPD	Laparoscopic pancreaticoduodenectomy
OPD	Open pancreaticoduodenectomy
SMA	Superior mesenteric artery
SMV	Superior mesenteric vein
PV	Portal vein
CHA	Common hepatic artery
PHA	Proper hepatic artery
CBD	Common bile duct
GDA	Gatroduodenal artery
MPD	Main pancreatic duct
PJ	Pancreaticojejunostomy
HJ	Hepaticojejunostomy
GJ	Gastrojejunostomy

## References

[1] Zureikat A.H., Beane J.D., Zenati M.S., Al Abbas A.I., Boone B.A., Moser A.J., Bartlett D.L., Hogg M.E., Zeh H.J. (2021). 500 Minimally Invasive Robotic Pancreatoduodenectomies: One Decade of Optimizing Performance. Ann. Surg..

[2] Palanivelu C., Senthilnathan P., Sabnis S.C., Babu N.S., Srivatsan Gurumurthy S., Anand Vijai N., Nalankilli V.P., Raj P.P., Parthasarathy R., Rajapandian S. (2017). Randomized clinical trial of laparoscopic versus open pancreatoduodenectomy for periampullary tumours. Br. J. Surg..

[3] Uijterwijk B.A., Wei K., Kasai M., Ielpo B., van Hilst J., Chinnusamy P., Lemmers D.H., Burdio F., Senthilnathan P., Besselink M.G. (2023). Minimally invasive versus open pancreatoduodenectomy for pancreatic ductal adenocarcinoma: Individual patient data meta-analysis of randomized trials. Eur. J. Surg. Oncol..

[4] Dokmak S., Ftériche F.S., Aussilhou B., Bensafta Y., Lévy P., Ruszniewski P., Belghiti J., Sauvanet A. (2015). Laparoscopic pancreaticoduodenectomy should not be routine for resection of periampullary tumors. J. Am. Coll. Surg..

[5] Gagner M., Pomp A. (1994). Laparoscopic pylorus-preserving pancreatoduodenectomy. Surg. Endosc..

[6] Boggi U., Amorese G., Vistoli F., Caniglia F., De Lio N., Perrone V., Barbarello L., Belluomini M., Signori S., Mosca F. (2015). Laparoscopic pancreaticoduodenectomy: A systematic literature review. Surg. Endosc..

[7] Mabrut J.Y., Fernandez-Cruz L., Azagra J.S., Bassi C., Delvaux G., Weerts J., Fabre J., Boulez J., Baulieux J., Peix J. (2005). Laparoscopic pancreatic resection: Results of a multicenter European study of 127 patients. Surgery.

[8] Napoli N., Kauffmann E.F., Palmeri M., Miccoli M., Costa F., Vistoli F., Amorese G., Boggi U. (2016). The Learning Curve in Robotic Pancreaticoduodenectomy. Dig. Surg..

[9] Giulianotti P.C. (2003). Robotics in General Surgery. Arch. Surg..

[10] Kornaropoulos M., Moris D., Beal E.W., Makris M.C., Mitrousias A., Petrou A., Felekouras E., Michalinos A., Vailas M., Schizas D. (2017). Total robotic pancreaticoduodenectomy: A systematic review of the literature. Surg. Endosc..

[11] Giulianotti P.C., Mangano A., Bustos R.E., Gheza F., Fernandes E., Masrur M.A., Gangemi A., Bianco F.M. (2018). Operative technique in robotic pancreaticoduodenectomy (RPD) at University of Illinois at Chicago (UIC): 17 steps standardized technique: Lessons learned since the first worldwide RPD performed in the year 2001. Surg. Endosc..

[12] Giulianotti P.C., Mangano A., Bustos R.E., Fernandes E., Masrur M.A., Valle V., Gangemi A., Bianco F.M. (2020). Educational step-by-step surgical video about operative technique in robotic pancreaticoduodenectomy (RPD) at University of Illinois at Chicago (UIC): 17 steps standardized technique—Lessons learned since the first worldwide RPD performed in the year 2001. Surg. Endosc..

[13] van Hilst J., Korrel M., de Rooij T., Lof S., Busch O.R., Groot Koerkamp B., Kooby D.A., van Dieren S., Abu Hilal M., Besselink M.G. (2019). Oncologic outcomes of minimally invasive versus open distal pancreatectomy for pancreatic ductal adenocarcinoma: A systematic review and meta-analysis. Eur. J. Surg. Oncol..

[14] Winer J., Can M.F., Bartlett D.L., Zeh H.J., Zureikat A.H. (2012). The current state of robotic-assisted pancreatic surgery. Nat. Rev. Gastroenterol. Hepatol..

[15] Asbun H.J., Moekotte A.L., Vissers F.L., Kunzler F., Cipriani F., Alseidi A., D’angelica M.I., Balduzzi A., Bassi C., Björnsson B. (2020). The Miami International Evidence-based Guidelines on Minimally Invasive Pancreas Resection. Ann. Surg..

[16] Sattari S.A., Sattari A.R., Makary M.A., Hu C., He J. (2023). Laparoscopic Versus Open Pancreatoduodenectomy in Patients with Periampullary Tumors: A Systematic Review and Meta-analysis. Ann. Surg..

[17] Papalampros A., Niehaus K., Moris D., Fard-Aghaie M., Stavrou G., Margonis A.G., Angelou A., Oldhafer K. (2016). A safe and feasible “clock-face” duct-to-mucosa pancreaticojejunostomy with a very low incidence of anastomotic failure: A single center experience of 248 patients. J. Visc. Surg..

[18] Klotz R., Mihaljevic A.L., Kulu Y., Sander A., Klose C., Behnisch R., Joos M.C., Kalkum E., Nickel F., Knebel P. (2024). Robotic versus open partial pancreatoduodenectomy (EUROPA): A randomised controlled stage 2b trial. Lancet Reg. Health Europe.

[19] Boone B.A., Zenati M., Hogg M.E., Steve J., Moser A.J., Bartlett D.L., Zeh H.J., Zureikat A.H. (2015). Assessment of quality outcomes for robotic pancreaticoduodenectomy: Identification of the learning curve. JAMA Surg..

[20] Speicher P.J., Nussbaum D.P., White R.R., Zani S., Mosca P.J., Blazer D.G., Clary B.M., Pappas T.N., Tyler D.S., Perez A. (2014). Defining the Learning Curve for Team-Based Laparoscopic Pancreaticoduodenectomy. Ann. Surg. Oncol..

[21] Zhao G., Liu Q., Zhao Z., Zhang X., Gao Y., Tan X., Liu R. (2022). The standardized technique and surgical video of robotic pancreaticoduodenectomy at the Chinese PLA General Hospital. Updates Surg..

